# Neurotransmitters as food supplements: the effects of GABA on brain and behavior

**DOI:** 10.3389/fpsyg.2015.01520

**Published:** 2015-10-06

**Authors:** Evert Boonstra, Roy de Kleijn, Lorenza S. Colzato, Anneke Alkemade, Birte U. Forstmann, Sander Nieuwenhuis

**Affiliations:** ^1^Cognitive Psychology Unit, Institute of Psychology, Leiden University, Leiden, Leiden; ^2^Leiden Institute for Brain and Cognition, Leiden University, Leiden, Leiden; ^3^Cognitive Science Center Amsterdam, University of Amsterdam, Amsterdam, Netherlands; ^4^Max Planck Institute for Human Cognitive and Brain Sciences, Leipzig, Germany

**Keywords:** GABA, cognition, blood–brain barrier, enteric nervous system, food supplements

## Abstract

Gamma-aminobutyric acid (GABA) is the main inhibitory neurotransmitter in the human cortex. The food supplement version of GABA is widely available online. Although many consumers claim that they experience benefits from the use of these products, it is unclear whether these supplements confer benefits beyond a placebo effect. Currently, the mechanism of action behind these products is unknown. It has long been thought that GABA is unable to cross the blood–brain barrier (BBB), but the studies that have assessed this issue are often contradictory and range widely in their employed methods. Accordingly, future research needs to establish the effects of oral GABA administration on GABA levels in the human brain, for example using magnetic resonance spectroscopy. There is some evidence in favor of a calming effect of GABA food supplements, but most of this evidence was reported by researchers with a potential conflict of interest. We suggest that any veridical effects of GABA food supplements on brain and cognition might be exerted through BBB passage or, more indirectly, via an effect on the enteric nervous system. We conclude that the mechanism of action of GABA food supplements is far from clear, and that further work is needed to establish the behavioral effects of GABA.

## Introduction

Gamma-aminobutyric acid (GABA) serves as the main inhibitory neurotransmitter in the human cortex (Roberts and Kuriyama, 1968; Petroff, 2002). In recent years it has become widely available as a food supplement. In Europe and the United States, GABA is considered a “food constituent” and a “dietary supplement,” respectively. As such, manufacturers are not required to provide evidence supporting the efficacy of their products as long as they make no claims with regards to potential benefits in relation to specific diseases or conditions. These GABA food supplements can be purchased online via numerous websites, including web shop giants such as Amazon.com, with often very positive customer reviews. Hundreds of people report that these supplements have helped them alleviate anxiety and/or improve sleep quality, in addition to other beneficial effects. Interestingly, GABA has long been thought to be unable to cross the blood–brain barrier (BBB), which raises questions about the mechanisms of action behind such beneficial effects (Roberts et al., 1958; Van Gelder and Elliott, 1958; Kuriyama and Sze, 1971; Knudsen et al., 1988). Through what mechanisms do these products exert their action? Do they rely on a placebo effect only? Do they exert an effect through peripheral effects outside of the brain? Or is GABA able to cross the BBB after all?

The current paper aims to give a succinct overview of recent understanding of GABA’s BBB permeability (Blood–Brain Barrier Permeability), the role of GABA in treatment of diseases (GABA, Diseases, and Treatment), its role as a food supplement (GABA as a Food Supplement), and the possibility that this food supplement might affect the central nervous system through an effect on the enteric nervous system (Enteric Nervous System Effects of GABA).

### Blood–Brain Barrier Permeability

The BBB protects most of the brain from toxins and ion abnormalities that find their way into vascular space through ingestion, infection, or other means (Purves et al., 2004). On the one hand, the BBB is important in keeping the brain safe from harmful substances. On the other hand it severely limits the passage of substances into the brain that might be beneficial to the individual, such as drugs to treat central nervous system disorders (Pardridge, 2005).

**FIGURE 1:**
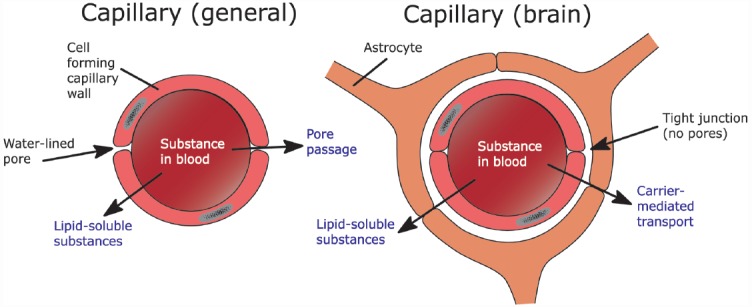
**The difference between capillaries as they are generally found in the body versus the ones in the brain and the possible ways for a substance to move across these capillaries**.

The BBB is made up by neighboring capillary endothelial cells. These cells are connected via tight junctions, which are impermeable (Brightman and Reese, 1969). As a consequence, molecules need to enter via active uptake by specialized transporter molecules or diffusion into the cells of the BBB (Pardridge, 2005, 2007). Tight junctions are responsible for the brain’s high resistance to outside materials. These tight junctions are not present in the rest of the body, where much more ionic and molecular traffic is possible (see Figure 1; Purves et al., 2004). As a consequence, the diffusion of a substance depends on its ability to cross the cell membrane, which consists largely of a lipid bilayer. The ability of a substance to pass through this lipid bilayer (i.e., its lipophilicity) depends largely on basic chemical properties (Lipinski, 2000; Pardridge, 2005).

Initial studies from the fifties reported GABA’s inability to cross the BBB (Van Gelder and Elliott, 1958). Since then, several research groups have replicated this finding (Roberts et al., 1958; Kuriyama and Sze, 1971; Knudsen et al., 1988). However, a number of studies have reported that GABA does cross the BBB, albeit in small amounts (Frey and Löscher, 1980; Löscher, 1981; Löscher and Frey, 1982; Al-Sarraf, 2002; Shyamaladevi et al., 2002). This discrepancy could be the result of variation in chemical compounds, method of administration (i.e., oral versus injection), and the species used.

**FIGURE 2:**
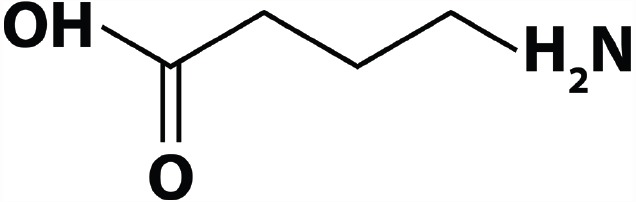
**GABA’s chemical structure**.

With regards to the first factor, not every study has employed the same chemical compound. One study administered 4-amino-3-hydroxybutyric acid (Kuriyama and Sze, 1971). Although this compound has a different chemical structure than GABA (i.e., an extra OH group), this study is often cited as providing evidence for GABA’s inability to cross the BBB. In view of the role that simple chemical properties play in BBB permeation, it might be problematic to generalize findings with different chemical compounds to GABA as it is found in the central nervous system and its food supplement version. All other studies that have reported evidence for or against GABA’s BBB permeability either administered radioactively labeled GABA (which is chemically identical to GABA, see Figure 2), or did not further specify the kind of GABA they used.

A second factor that may, in principle, account for the discrepancy between animal studies concerns the significant variation in methods of GABA administration. GABA was administered either by intraperitoneal injection (Van Gelder and Elliott, 1958; Kuriyama and Sze, 1971; Frey and Löscher, 1980; Löscher, 1981; Shyamaladevi et al., 2002), intravenous injection (Roberts et al., 1958; Löscher and Frey, 1982; Knudsen et al., 1988), or the bilateral *in situ* brain perfusion technique (Al-Sarraf, 2002). However, there appears to be no systematic relationship between the method of administration and the research outcome; positive and negative evidence has been found with all of these methods.

Thirdly, the reported studies differ in the species of animals tested. Most studies used rats (Van Gelder and Elliott, 1958; Kuriyama and Sze, 1971; Al-Sarraf, 2002; Shyamaladevi et al., 2002), but mice (Roberts et al., 1958; Frey and Löscher, 1980), rabbits (Van Gelder and Elliott, 1958; Kuriyama and Sze, 1971), and dogs (Löscher and Frey, 1982) have also been used. As with the employed methodologies, both positive and negative evidence has been found with these different species.

One limitation of this field is that there have been no studies with humans that directly assessed GABA’s BBB permeability. This is not so surprising given the limited number of methods for measuring GABA levels in the human brain. GABA levels have been determined in post-mortem tissue samples (Perry et al., 1973). Additionally, neocortical slices have been extracted from epileptic patients undergoing surgery (Errante et al., 2002), but these methods have not been employed to assess the effect of GABA administration on brain GABA levels. The obvious noninvasive candidate for such an assessment is magnetic resonance spectroscopy (MRS), but we are not aware of any MRS studies that assessed brain GABA levels after administration of GABA. Assessment of GABA concentrations in the brain using MRS requires a careful experimental design, since GABA is not only present in the brain, but also in blood vessels located outside of the BBB. Tissue fraction analyses estimating blood, CSF, gray matter and white matter presence within each volume of interest should therefore be incorporated (Draper et al., 2014).

Interestingly, evidence has been found for the presence of a GABA-transporter in the BBB (Takanaga et al., 2001). The expression of such a transporter indicates that GABA can enter and/or exit the brain through facilitated transport. In mice, the brain efflux rate for GABA was found to be 17 times higher than the influx rate (Kakee et al., 2001). This complicates the interpretation of GABA concentrations in the brain, and it is possible that this may have led to an underestimation of the extent to which GABA is able to cross the BBB. That is, some studies may have found little evidence for GABA’s BBB permeability because of the high efflux rate.

### GABA, Diseases, and Treatment

Increasing GABA in the brain has for years been the focus of drug development aiming to alleviate the severity of epileptic seizures (Hawkins and Sarett, 1957; Wood et al., 1979; Gale, 1989; Petroff et al., 1995). Initial studies examined the efficacy of administering GABA directly. One study reported a reduction in the amount of seizures in epileptic patients who were administered a very high dose of GABA (0.8 g/kg daily; Tower, 1960). However, this result was found only in four out of twelve patients. Additionally, the patients in whom the administration of GABA did have an effect were children below the age of 15. This finding is in line with the suggestion that the BBB permeability to GABA decreases with age (Al-Sarraf, 2002). Perhaps more importantly, GABA’s half-life is about 17 min in mice (Kakee et al., 2001). If the half-life has a similar short duration in humans, direct administration of GABA is unsuitable as pharmacological treatment of epilepsy.

The GABA analog gabapentin was developed as an anti-epileptic drug. Gabapentin functions by modulating enzymes involved in GABA synthesis. It differs in chemical structure from GABA and its half-life is much longer (McLean, 1994). One MRS study in humans has found that the administration of gabapentin increased brain GABA levels by 55.7% (Cai et al., 2012). Nonetheless, a study exploring the effects of gabapentin in both rat and human neocortical slice preparations suggests that there might be a considerable difference between rodents and humans in the effects on GABA levels: gabapentin was found to increase GABA concentrations by 13% in human neocortical slices, while having no significant effect in rat neocortical slices (Errante et al., 2002).

Patients with Huntington’s disease also have reduced GABA levels in the brain (Perry et al., 1973), but administration of GABA to remedy this deficiency has shown mixed results with regards to the reduction of symptoms (Barbeau, 1973; Fisher et al., 1974; Shoulson et al., 1976). Of course, that the administration of GABA does not consistently alter the symptoms in complex and multifaceted disorders such as epilepsy and Huntington’s disease, does not necessarily mean that GABA is unable to affect the brain.

### GABA as a Food Supplement

In recent years researchers have reported a number of placebo-controlled studies in which GABA was administered as a food supplement to healthy participants and participants with a history of acrophobia. One study found an increase in alpha waves in healthy participants and reduced levels of immunoglobulin A (IgA; an indicator of immune system functioning) in participants with a history of acrophobia when they were exposed to heights (Abdou et al., 2006). However, the sample size for the second finding was very small (four participants per group). Another study reported reduced heart rate variability and salivary chromogranin A (CgA) during an arithmetic task compared to a control group after the administration of GABA-enriched chocolate (Nakamura et al., 2009). A third study reported less salivary cortisol and CgA than a control group during a psychological stress-inducing arithmetic task. Additionally, participants who received 50 mg of GABA dissolved in a beverage reported less psychological fatigue after completion of the task (Kanehira et al., 2011). Finally, in a fourth study, participants were found to show a decrease in alpha waves over time while performing an arithmetic task. This decrease was smaller in the group that orally received GABA (100 mg) compared to a control group (Yoto et al., 2012). By way of comparison, one would have to eat 2.34 kg of uncooked spinach in order to consume a similar amount of GABA, and spinach is relatively rich in GABA compared to other foods (Oh et al., 2003).

The results of these studies support the claims made by hundreds of consumers of GABA food supplement products and fit with a growing trend in which GABA is administered through everyday (natural) foods (Diana et al., 2014). However, there are some caveats to consider. First, at least one of the authors in each of these four studies was affiliated with the company that produces the GABA supplement in question. However, a declaration of conflicting interests is lacking in three out of four of these studies. Second, the reported studies used “pharma-GABA,” which is produced for the Asian market through a fermentation process using a strain of lactic acid bacteria, *Lactobacillus hilgardii K-3* (Kanehira et al., 2011). Pharma-GABA has been approved by the FDA as a food ingredient (Food and Drug Administration, 2008). While the manufacturer of pharma-GABA suggests that there are important differences with the synthetic GABA supplement sold online in Western countries (http://www.natural-pharmagaba.com/q-and-a.html), these differences refer to the production process and the occurrence of potentially harmful byproducts in synthetically produced GABA, and not to the chemical structure of the active compound GABA.

A recent study by Steenbergen et al. (2015a) with human subjects has shown that the ingestion of synthetic GABA (800 mg) enhanced the ability of prioritized planned actions and inhibitory control (as indexed by the stop-change task; Verbruggen and Logan, 2008; Steenbergen et al., 2015a). However, in view of the lack of evidence with regards to GABA’s BBB permeability in humans, the mechanism through which GABA might have exerted these effects remains unclear. The same holds for the pharma-GABA studies that were discussed above: none of these effects exclude an indirect of GABA on the brain. The oral intake of these supplements may have exerted these effects through indirect pathways, for example through the enteric nervous system (ENS).

### Enteric Nervous System Effects of GABA

The bidirectional signaling between the brain and the ENS is vital in maintaining homeostasis (Cryan and O’Mahony, 2011). Even though most research thus far has focused on the signaling from the brain to the gut, an increasing number of studies has explored the influence of the gut’s microbiota on the brain. For example, gut microbiota have been shown to improve mood and reduce anxiety in patients with chronic fatigue (Logan and Katzman, 2005; Rao et al., 2009). Similarly, oral intake of probiotics resulted in reduced urinary cortisol and perceived psychological stress (Messaoudi et al., 2011) and reduced reactivity to sad mood (Steenbergen et al., 2015b) in healthy subjects.

It has been found that certain probiotic strains are able to produce GABA *in vivo*. Specifically, bacteria from the strains *Lactobacillus* and *Bifidobacterium* were effective at increasing GABA concentrations in the ENS (Barrett et al., 2012). Indeed, both GABA and its receptors are widely distributed through the ENS (Auteri et al., 2015). Additionally, there is considerable communication between the gut and the brain through the vagal nerve (Cryan and O’Mahony, 2011; Cryan and Dinan, 2012). This nerve consists, for the most part, of sensory nerve fibers that relay information about the state of bodily organs to the central nervous system (Thayer and Sternberg, 2009).

A study in mice showed that the administration of *Lactobacillus rhamnosus* (JB-1) consistently modulated the mRNA expression of GABA_Aα2_, GABA_Aα1_, and GABA_B1b_ receptor subunits (Bravo et al., 2011), receptors commonly associated with anxiety-like behavior. Indeed, on a behavioral level the *L. rhamnosus* (JB-1)-fed mice were less anxious and displayed antidepressant-like behaviors in comparison with controls. Furthermore, the administration of these bacteria reduced the stress-induced elevation of corticosterone compared to the control mice. Importantly, none of these effects were present in mice that underwent vagotomy (Bravo et al., 2011).

In humans, the stimulation of the vagus nerve through transcutaneous vagus nerve stimulation (tVNS) has been used to treat refractory epilepsy (Vonck et al., 2014). This technique has been shown to affect norepinephrine, acetylcholine and GABA concentrations (Van Leusden et al., 2015). With regards to GABA, VNS seems to increase the level of free GABA in the cerebrospinal fluid (Ben-Menachem et al., 1995). Similarly to the administration of synthetic GABA (Steenbergen et al., 2015a), active tVNS was found to enhance the ability of prioritizing and cascading different actions when performing a stop-change paradigm (Steenbergen et al., 2015c).

To summarize, bacteria from the *Lactobacillus* spp. strain contribute to the formation of GABA in the ENS. The oral administration of bacteria from this strain can influence GABAergic firing in the mice brain through the vagus nerve. Furthermore, stimulation of the vagal nerve through tVNS has been shown to affect processes thought to be GABAergic in humans. Finally, a similar behavioral effect has been found both for the administration of synthetic GABA and tVNS with regards to action cascading. Even if GABA is unable to cross the BBB at all in humans, an indirect effect through the ENS might be a viable route for an effect of GABA food supplements. The link between the oral administration of GABA, the vagal nerve and GABA levels in the brain has not been established yet, but in view of the available evidence it is a promising candidate for future research.

## Conclusion

In this paper we have discussed the conflicting evidence with regards to GABA’s BBB permeability. There are both a number of studies that were unable to show that GABA crosses the BBB and a number of studies that did show GABA’s ability to cross. In view of the multitude of employed methods and species, in addition to the finding that GABA metabolism might differ between rodents and humans (Errante et al., 2002), it is not possible at this time to come to a definite conclusion with regards to GABA’s BBB permeability in humans. The mixed findings concerning GABA administration in clinical populations suffering from epilepsy or Huntington’s disease are insufficient to rule out a possible effect of GABA in the brain. Perhaps the amount of GABA that reaches the brain is too small to be of clinical significance, but large enough for an effect in a stop-change paradigm. We believe that MRS studies are the most promising technique to directly assess the effect of GABA administration on GABA levels in the human brain. Interestingly, in one of the discussed studies with rats, GABA by itself was found to increase brain GABA by 33%, but when GABA was administered together with L-arginine, brain GABA increased by 383.3% (Shyamaladevi et al., 2002). The authors suggest that this dramatic increase in brain GABA might be caused by an L-arginine-mediated increase in nitric oxide, which is thought to affect BBB permeability (Shukla et al., 1996). It would be interesting to see if this effect can be replicated in humans.

Furthermore, we discussed GABA’s role as a food supplement and the way in which these products might exert an effect other than through BBB permeation. There is some evidence for the claims made by hundreds of consumers online concerning the calming effects of GABA food supplements, but evidence from independent studies is needed. In addition, even if a calming effect of GABA can be reliably demonstrated, the mechanism through which these supplements work is unclear. We have suggested that GABA supplements might work through the ENS, but far more research is needed in order to support this hypothesis. Indeed, at this point it is even too early to conclude whether these supplements reach the brain in sufficient concentrations to exert a biologically relevant effect.

### Conflict of Interest Statement

The authors declare that the research was conducted in the absence of any commercial or financial relationships that could be construed as a potential conflict of interest.
